# Super-Cationic Peptide Dendrimers—Synthesis and Evaluation as Antimicrobial Agents

**DOI:** 10.3390/antibiotics10060695

**Published:** 2021-06-10

**Authors:** Estelle J. Ramchuran, Isabel Pérez-Guillén, Linda A. Bester, René Khan, Fernando Albericio, Miguel Viñas, Beatriz G. de la Torre

**Affiliations:** 1 Biomedical Resource Unit, School of Laboratory Medicine and Medical Sciences, College of Health Sciences, University of KwaZulu-Natal, Durban 4000, South Africa; 209534898@stu.ukzn.ac.za (E.J.R.); Besterl@ukzn.ac.za (L.A.B.); 2 Peptide Sciences Laboratory, School of Chemistry and Physics, University of KwaZulu-Natal, University Road, Westville, Durban 4001, South Africa; 3 Lab Molecular Microbiology & Antimicrobials, Department of Pathology and Experimental Therapeutics, Medical School-IDIBELL, University of Barcelona, Hospitalet, 08907 Barcelona, Spain; isaperezguillen@gmail.com; 4 Discipline of Medical Biochemistry, School of Laboratory Medicine and Medical Science, University of KwaZulu-Natal, Durban 4001, South Africa; Myburgr@ukzn.ac.za; 5 Institute for Advanced Chemistry of Catalonia (IQAC-CSIC), 08034 Barcelona, Spain; 6 CIBER-BBN, Networking Centre on Bioengineering, Biomaterials and Nanomedicine, Department of Organic Chemistry, University of Barcelona, 08028 Barcelona, Spain; 7 KRISP, College of Health Sciences, University of KwaZulu-Natal, Westville, Durban 4001, South Africa

**Keywords:** solid-phase peptide synthesis, antimicrobial peptides, Gram-positive, Gram-negative, therapeutic index

## Abstract

Microbial infections are a major public health concern. Antimicrobial peptides (AMPs) have been demonstrated to be a plausible alternative to the current arsenal of drugs that has become inefficient due to multidrug resistance. Herein we describe a new AMP family, namely the super-cationic peptide dendrimers (SCPDs). Although all members of the series exert some antibacterial activity, we propose that special attention should be given to (KLK)_2_KLLKLL-NH_2_ (G1KLK-L2KL2), which shows selectivity for Gram-negative bacteria and virtually no cytotoxicity in HepG2 and HEK293. These results reinforce the validity of the SCPD family as a valuable class of AMP and support G1KLK-L2KL2 as a strong lead candidate for the future development of an antibacterial agent against Gram-negative bacteria.

## 1. Introduction

The emergence and spread of multidrug-resistant (MDR) bacteria have become a major challenge for antimicrobial therapy [1]. Indeed, multidrug resistance is a major public health issue [2]. The scenario has become increasingly more alarming given the large gap between the development of novel antibiotics and the burden of infections due to MDR bacteria [3]. Thus, research into new antimicrobial products and strategies is urgent [4].

Antimicrobial peptides (AMPs), either natural products produced by microorganisms, animals, plants and bacteria, or synthetic ones, are one of the main options to overcome bacterial drug resistance [5]. In principle, this is based on the fact that they are potent antimicrobials and they do not share mechanisms of action with known traditional antibiotics. Moreover, some peptides, particularly those similar to animal defense AMPs, have immunomodulatory properties [6]. Widespread resistance, including extremely resistant microorganisms, together with the lack of new antimicrobial agents, has led to the revival of abandoned antimicrobials, such as colistin, a natural AMP, and has driven research into the synthesis of similar molecules [7], as well as the search for new ones [8,9]. In general, AMPs have a positive net charge that allows them to interact selectively with bacterial membranes and also with other negatively charged structures, including DNA, lipopolysaccharide (LPS) and lipoteichoic acids (LTAs). Although membrane permeabilization seems to be the main mechanism of action of AMPs, a few additional mechanisms have also been reported, such as inhibition of the biosynthesis of macromolecules, inhibition of nucleic acid synthesis and alterations in intracellular translocation and in metabolism. Transcriptomics studies have revealed that AMPs commonly lead to a dramatic alteration of bacterial gene expression. Surprisingly, the toxicity of AMPs on eukaryotic cells in vitro is generally low.

Numerous AMPs, including linear alpha helical amphipathic peptides [10] and also peptidomimetics such as peptoids [11], foldamers [12] and other amide-containing oligomers [13], act by disrupting microbial membranes. Peptides based on a dendrimer structure are an interesting case and could be considered multiple ramified peptides through a branched unit, which is usually a Lys residue. This concept has been explored by Reymond et al. using mostly units based on Leu-Lys [14,15,16]. In the study herein, we examined the antimicrobial activity of different generations of super-cationic peptide dendrimers based on the triad XLX, where X are residues of Lys, Orn or Arg.

## 2. Results and Discussion

### 2.1. Design and Synthesis of Super-Cationic Peptide Dendrimers (SCPDs)

Molecule design was based on the following three main physical driving forces behind the antibacterial activity of cationic AMPs: (i) highly positive charge to enhance interaction with the anionic lipids of the cell wall in bacteria, (ii) hydrophobic content to facilitate membrane insertion and (iii) flexibility to allow conformational changes when interacting with membranes [17,18]. With this in mind, two “mirror sequences” were used: LLKLL for G0, which has a high hydrophobic content, and XLX for the branches of the following generations, where X is a basic residue, and hence this sequence contributes two positive charges to each branch. The branching is afforded by the incorporation of a Lys residue, which, after reaction on the α- and ε- amino groups, can be considered as contributing to the hydrophobicity of the molecule. Moreover, we included a series of compounds with N-terminals acylated with different lengths of the acylating moiety (acetyl, hexyl and dodecyl), which directly affects the hydrophobicity of the resulting molecule (Table 1).

**Figure 1:**
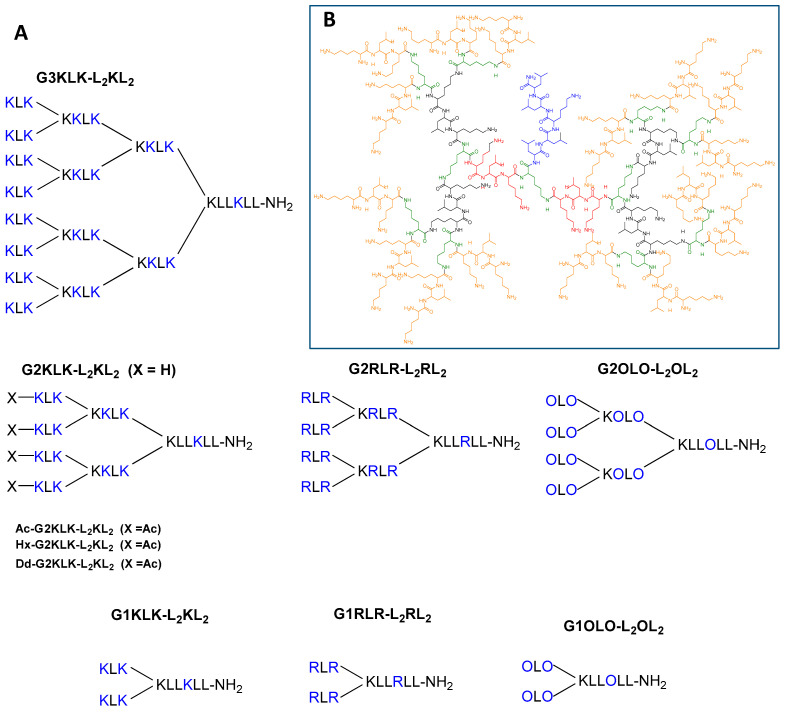
(**A**) Schematic representation of the peptide dendrimers synthesized. (**B**) 2-D representation of G3KLK-L_2_KL_2_; the green residues are the branching Lys, then blue, red, black and orange indicate G0, G1, G2 and G3, respectively.

All the constructs were synthesized by standard solid-phase peptide synthesis (SPPS) following the Fmoc/tBu methodology. They were then purified to >95% homogeneity by reverse-phase HPLC and characterized by LCMS. The schematic representation of the 10 compounds is given in Figure 1A, and a 2-D representation of G3KLK-L_2_KL_2_ is shown in Figure 1B.

### 2.2. Antimicrobial Action (MIC and MBC)

The antimicrobial activity of the 10 peptide dendrimers described before was first assayed against a panel of antibiotic-susceptible reference bacterial strains (three Gram-positive and two Gram-negative). The MIC (minimum inhibitory concentration) and MBC (minimum bactericidal concentration) values ranged from 8 to 256 µg/mL, as can be seen in Table 2.

It is acknowledged that there is a poor understanding of why the bacteria in vitro susceptibility against an antibacterial agent results in pitfalls when tested in clinical infections. The MIC determination only concerns two factors, bacteria and drug while in clinical infection, but there is an extra factor to be considered: the patient. To simulate an environment more realistic, the MICs are determined by adding 50% of human serum to the testing media. In fact, MIC values, as well as time–kill studies, of some compounds can present significant modifications in their antimicrobial capability when tested in the presence of human serum. This is the case of Mu1140, a peptide produced by *Lactococcus lactis*; its MIC increased up to fourfold when tested against *S. pneumoniae* in the presence of human serum, likely because the antibiotic binding to serum proteins results in a lower effective concentration. In contrast, the opposite outcome was seen against *S. aureus*—MIC decreases to one-fourth [17]. In our study, the addition of 50% human serum had no effect on MIC values in any of the bacterial strains tested.

The MIC values appear to indicate that the tested compounds are broad-spectrum antimicrobials since they exerted activity against both Gram-positive and -negative strains. Only compound G1KLK-L_2_KL_2_ showed a certain degree of selectivity against Gram-negative strains. In spite of the fragility of the information provided by MIC values, a comparison of the compounds indicated that (i) the different levels of branching does not significantly affect antimicrobial activity against the bacterial strains tested, and (ii) the acylation of the N-termini does not contribute to improving this activity. In contrast, a longer acyl chain was revealed as being detrimental, giving higher MIC values. In this regard, the peptide dendrimer Dd-G2KLK-L_2_KL_2_ was discarded for further experiments.

Comparison of the MBC and MIC values revealed that they were very close, in most cases differing in one level of dilution, which is insignificant. This finding would suggest that this family of peptide dendrimers exert bactericidal activity. To confirm this hypothesis, time–kill kinetics and growth curve studies were conducted, and they are discussed in a further section.

Next, the antimicrobial action of the compounds was tested on resistant strains: MRSA (methicillin-resistant *S. aureus,* Table 3), VRE (vancomycin-resistant *Enterococcus,* Table 4) and CRE (carbapenem-resistant Enterobacteriaceae, Table 5). As expected, antimicrobial activity against resistant isolates did not differ significantly from the values measured in susceptible strains. This observation suggests that although bacterial resistance to SCPDs cannot be ruled out, the mechanisms of such resistance would be different to those determining resistance to conventional antibiotics.

As reflected by the MIC values, the SCPDs synthesized apparently work as broad-spectrum antimicrobials since they show activity against both Gram-positive and -negative bacterial strains. The observation that the MIC values are very close for the two types of bacteria would suggest no differences in the mode of action exerted by the compounds. Thus, this is an indication that permeability is not an obstacle for SCPD action. In principle, the outer membrane of Gram-negative bacteria is a permeability barrier and a first obstacle preventing antimicrobials from exerting their action.

Furthermore, it is relevant to bear in mind that vancomycin is the last resort to treat MRSA infections as it is nephrotoxic, inducing interstitial nephritis and tubular cell damage. Furthermore, vancomycin resistance is spreading. In this context, there is an urgent need to explore new, less toxic compounds with activity against MRSA as alternatives to vancomycin. In this regard, SCPDs emerge as promising molecules since they also covered vancomycin-resistant infections.

### 2.3. Cytotoxicity

**Figure 2:**
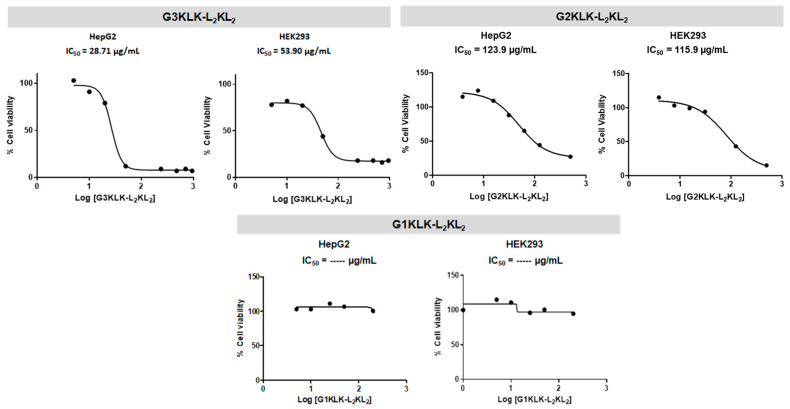
Cytotoxicity of G3KLK-L_2_KL_2_, G3KLK-L_2_KL_2_ and G3KLK-L_2_KL_2_ in HepG2 and HEK293 cell lines.

To assess the toxicity of the synthesized peptide dendrimers on eukaryotic cells, the 3-(4,5-dimethylthiazol-2-yl)-2,5-diphenyltetrazolium bromide (MTT) assay was used in two cell lines: HepG2 (human liver cancer cell line) and HEK293 (line derived from human embryonic kidney cells). The most branched peptide, G3KLK-L_2_KL_2_, showed the highest toxicity, with an IC_50_ of 25.7 and 53.9 µg/mL for HepG2 and HEK293, respectively (Figure 2). Compared with the MIC for this compound, these viability values make it unsuitable as a therapeutic application. On the other hand, the least branched peptide consisting of Lys as basic residues (G1KLK-L_2_KL_2_) did not show toxicity at the range of concentrations tested.

**Figure 3:**
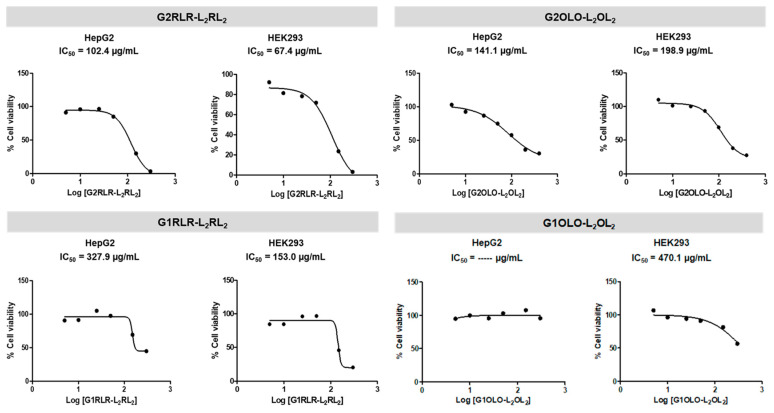
Cytotoxicity of G2RLR-L_2_RL_2_, G2OLO-L_2_OL_2_, G1RLR-L_2_RL_2_ and G1OLO-L_2_OL_2_ in HepG2 and HEK293 cell lines.

The compounds included in the G2 group (G2KLK-L_2_KL_2_, G2RLR-L_2_RL_2_, G2OLO-L_2_OL_2_) showed less toxicity than the G3 one. All the IC_50_ values for the G2 group were 3 to 4 times higher than the corresponding MIC. However, of note, the lower cell viability was exhibited by the compounds consisting of Arg as basic residues, especially in the case of HEK293 cells (Figure 3). The improvement of cell viability testing the G1 group, with one less branching level, was significant. Moreover, a similar behavior was observed in this group, the Arg-containing compounds once again being the most toxic.

### 2.4. Time–Kill Curves

Another approach to evaluate the antibacterial effect of one molecule is to consider the killing kinetics or time–kill curves on standardized cultures of bacteria. We did this against a Gram-positive strain, *S. aureus*, for compounds G2RLR-L_2_RL_2_, G2OLO-L_2_OL_2_ and G1OLO-L_2_OL_2_ as representative of those with the lowest MIC and highest IC_50_ values and thus with a potentially better therapeutic index. We also tested these compounds against two Gram-negative strains (*E. coli* and *P*. *aeruginosa*). In this case, we also included G1KLK-L_2_KL_2,_ which showed some selectivity against Gram-negative bacteria and low toxicity in eukaryotic cells.

**Figure 4:**
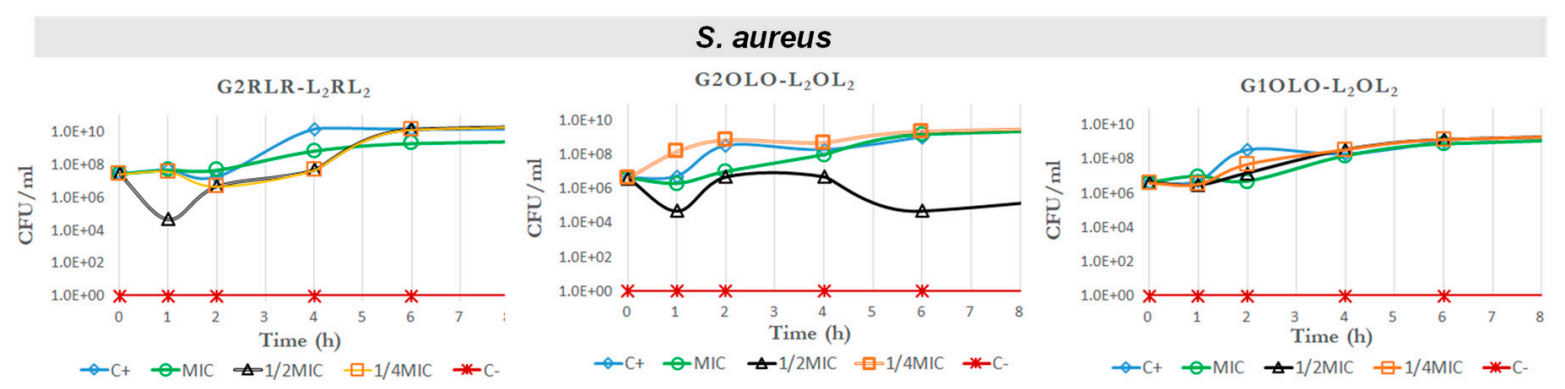
Time–kill curves against *S. aureus* for G2RLR-L_2_RL_2_, G2OLO-L_2_OL_2_ and G1OLO-L_2_OL_2_.

The time–kill curves for *S. aureus* (Figure 4) revealed that the three compounds had no effect on bacterial growth at MIC, ½ MIC and ¼ MIC, showing approximately the same behavior as the positive control at all the concentrations. These results seem to not be in agreement with the MIC values obtained, where these compounds showed an antibacterial effect at concentrations between 32 and 64 µg/mL. Nevertheless, the differences between quantitative results between MIC determinations and growth curves in the presence of antibacterials should be regarded as a result of the methods themselves. The ability of bacterial growth in a shaken 10 mL container (the conditions in which the growth curves are obtained in mini-fermenters) is much more favorable for the bacterium than microplate wells. Thus, it is reasonable that some differences in the concentrations to inhibit growth may differ between both culture conditions. On the other hand, the time–kill curves have been run at below MIC concentrations.

**Figure 5:**
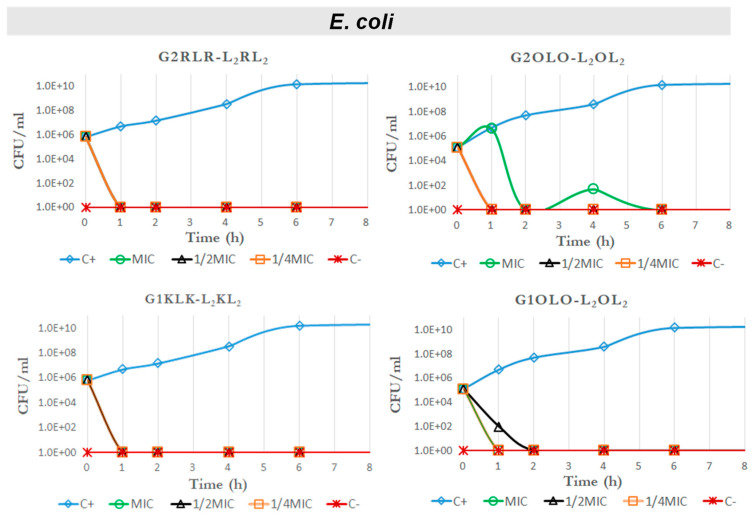
Time–kill curves against *E. coli* for G2RLR-L_2_RL_2_, G2OLO-L_2_OL_2_, G1KLK-L_2_KL_2_ and G1OLO-L_2_OL_2_.

In contrast, the curves for *E.coli* (Figure 5) showed that all the tested compounds killed the bacteria in less than 1 h at MIC, ½ MIC and ¼ MIC, and no regrowth was seen after 24 h (Figure S1, SI).

This devastating action of all four compounds even at ¼ MIC prompted us to examine the potential therapeutic index (TI) for each one, considering this concentration and toxicity (IC_50_) in HepG2 and Hek293 cell lines. In this regard, we found that G2RLR-L_2_RL_2_ would have a TI of approximately 6 and 4, respectively, which are not acceptable values for therapeutic applications. Compound G2OLO-L_2_OL_2_ showed better results, with a TI of 70 and 100 for HepG2 and Hek293 cells, respectively. These values could allow this compound to be studied further. Finally, the compounds G1KLK-L_2_KL_2_ and G1OLO-L_2_OL_2_ did not show toxicity for the two cell lines. The TI could be determined only for G1OLO-L_2_OL_2,_ which had an index of 235 for the kidney cell line. On the basis of our results, G1KLK-L_2_KL_2_ and G1OLO-L_2_OL_2_ emerge as the best candidates as new antimicrobial agents against *E. coli*.

**Figure 6:**
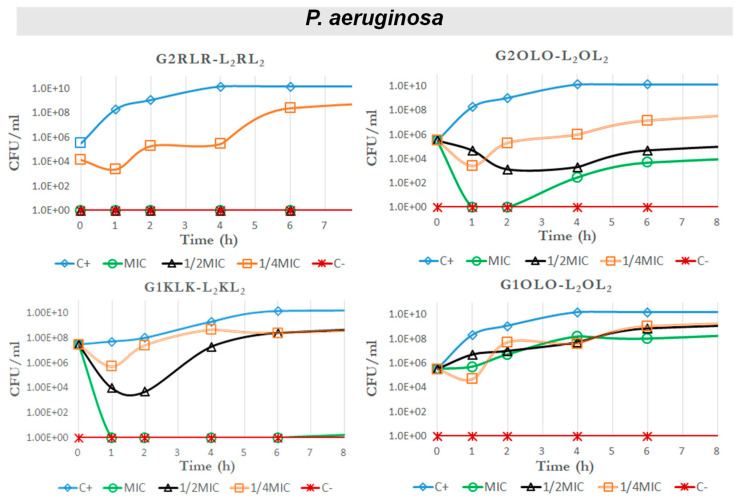
Time–kill curves against *P. aeruginosa* for G2RLR-L_2_RL_2_, G2OLO-L_2_OL_2_, G1KLK-L_2_KL_2_ and G1OLO-L_2_OL_2_.

In another Gram-negative bacteria, *P. aeruginosa* (Figure 6), a bacterial species characterized by intrinsic resistance to many antimicrobials, the action of the tested compounds was less dramatic that in previous case. Nevertheless, at MIC and ½ MIC, compound G2RLR-L_2_RL_2_ reduced the bacterial population to almost zero and no regrowth was appreciated after 24 h. However, at ¼ MIC, the bacterial population remained partially inhibited at the beginning of the exposure. At the MIC, G2OLO-L_2_OL_2_ killed all bacteria within the first hour of exposure and regrowth was observed after the second hour. A similar pattern was seen when this compound was tested at ½ MIC and ¼ MIC, although the bacterial population was not totally eliminated in these cases. After the second hour onwards, the bacterial population started to grow, but at a slower rate than in the control. This observation may suggest that, at low concentrations, G2OLO-L_2_OL_2_ acts as a bacteriostatic agent. A delay in antimicrobial action was also observed at ½ MIC in comparison with ¼ MIC. This phenomenon has been referred to as a paradoxical effect (or Eagle effect) [18]. In the case of G1KLK-L_2_KL_2_, all bacteria were eliminated after the first hour of exposure at the MIC and no regrowth was detected in the following 24 h. When ½ MIC was used, a reduction in bacteria of four orders of magnitude was observed after 1 h, but bacteria showed regrowth later on. The bacteria showed the same behavior in response to ¼ MIC, but the reduction was only two magnitudes of order. Finally, G1OLO-L_2_OL_2_ had no effect at any concentration tested.

Regarding the TIs, again as in the case of *E. coli*, G2RLR-L_2_RL_2_ gave small values and were lower (<2) in the case of *P. aeruginosa*, meaning this compound will have very poor therapeutic applicability. G2OLO-L_2_OL_2_ showed TI values (TI = 35 for HepG2 and TI = 50 for Hek293) that could be considered for therapeutic use, although the killing effect was observed only for a limited time. G1KLK-L_2_KL_2_ is the compound of choice since it showed the lowest cytotoxicity.

### 2.5. Effects on Growth Kinetics

**Figure 7:**
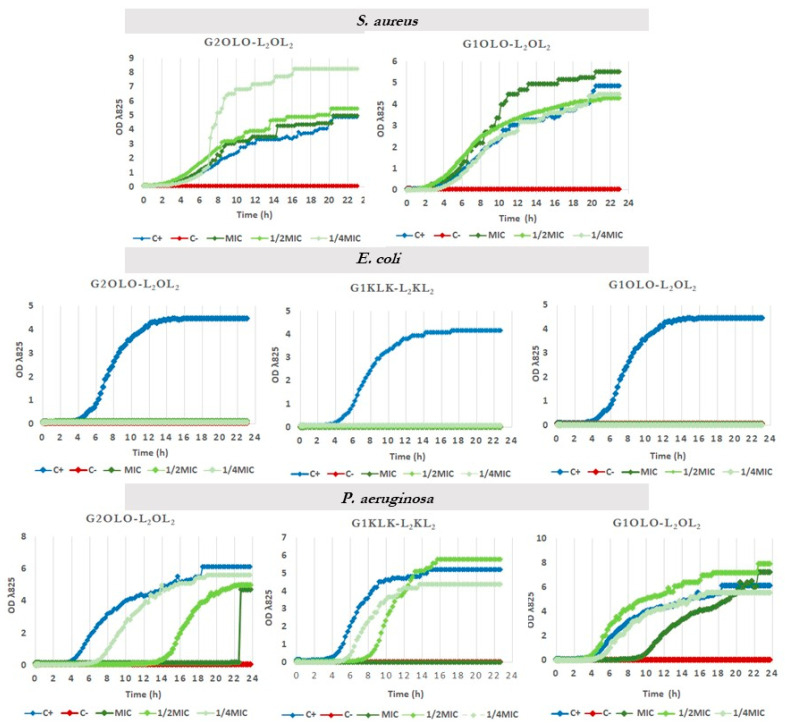
Growth curves of *S. aureus, E. coli* and *P aeruginosa* in the presence of MIC, ½ MIC and ¼ MIC of G2OLO-L_2_OL_2_ and G1OLO-L_2_OL_2_.

The two compounds based on Orn as basic residues, G2OLO-L_2_OL_2_ and G1OLO-L_2_OL_2_, and G1KLK-L_2_KL_2_ as the most promising, were assayed for their effect on the growth curve of the same bacterial strains used in the time–killing study. The effect of these compounds on *S. aureus* was negligible (Figure 7, upper panel) at MIC, ½ MIC and ¼ MIC. This lack of effect confirmed the results of the time–kill kinetics experiments shown above and are in disagreement with the values shown in MIC determinations. The assay in *E. coli* revealed that the three compounds abolished bacterial growth (Figure 7, central panel), irrespective of the concentration used (MIC, ½ MIC and ¼ MIC). These results also are in agreement with the observations made in the time–killing study. In *P. aeruginosa* (Figure 7, lower panel), G2OLO-L_2_OL_2_ abolished growth only at the MIC, and caused a 10 h delay in growth at ½ MIC and about a 2 h delay at ¼ MIC. These findings thus reveal a concentration-dependent effect. A similar effect was observed for G2KLK-L_2_KL_2_, but this compound showed less capacity to delay growth when the bacteria were exposed to ½ MIC and ¼ MIC. When the bacteria were treated with G1OLO-L_2_OL_2_ at the MIC, a delay of approximately 6 h in growth was observed, while at ½ MIC or ¼ MIC, there was no effect.

## 3. Conclusions

The search for new antimicrobial agents to fight bacterial resistance is a challenge. Here, we designed a set of branched peptides to obtain constructs that we have called super-cationic peptide dendrimers (SCPDs) and tested them as potential antimicrobial agents. The first screening determined their MIC and MBC values, revealing that almost all the compounds showed some antibacterial activity, with no significant differences between Gram-positive and Gram-negative microorganisms. Thus, these SCPDs appear to be broad-spectrum antibacterial compounds, except G1KLK-L_2_KL_2_, which was Gram-negative selective. Nevertheless, further testing, i.e., time–kill kinetics and growth curves, revealed considerable differences in the action, showing higher activity against Gram-negative bacteria, especially *E. coli*. On the other hand, the cytotoxicity study in HepG2 and HEK293 cell lines showed higher toxicity for the higher branched peptides. Although some of the compounds showed good antibacterial activity, the calculated TI make them unsuitable as therapeutic agents. However, this was not the case for compound G1KLK-L2KL2, whose TI makes it a strong lead candidate for the future development of an antibacterial agent against Gram-negative bacteria.

## 4. Materials and Methods

### 4.1. Chemical Synthesis

The SCPDs were synthesized manually by solid phase peptide synthesis methodology using Fmoc/*t*Bu strategy [19]. Briefly, Fmoc-Rink-amide-resin (0.76 mmol/g) was used in combination with Fmoc-Leu-OH, Fmoc-Arg(Pbf)-OH, Fmoc-Orn(Boc)-OH and Fmoc-Lys(Boc)-OH for the units and Fmoc-Lys(Fmoc)-OH for the branching (5 equiv. for each coupling). *N,N′-*diisopropylcarbodiimide (DIC) and OxymaPure (5 equiv. each) were used as coupling reagents and *N,N-*dimethylformamide (DMF) as a solvent for a 30 min reaction. Fmoc was removed by two treatments with piperidine-DMF (2:8) for 1 and 5 min. At the end of the synthesis, the dendrimers were detached from the resin by treatment with trifluoroacetic acid (TFA)-triisopropylsilane (TIS)-H_2_O (95:2.5:2.5) for 1 h. The filtrates were collected in cold diethyl ether (DEE) and the precipitated was isolated by centrifugation and decantation. The solid was washed three times with DEE, dissolved in 10% acetic acid and lyophilized. All peptides were purified using C18 reverse phase high-performance liquid chromatography (HPLC) and characterized by liquid chromatography-mass spectrometry (LC-MS) (see SI).

### 4.2. Bacterial Strains, Media and Antimicrobial Substances

Nunclon Delta Surface sterile microtiter plates (including the Edge 2.0 plate) and fetal calf serum were purchased from Thermo Fisher Scientific (Waltham, MA, USA). Human serum from male AB plasma (sterile and filtered), antibiotics, antimycotic solution, methylthiazol terazolium salt and all other reagents were obtained from Sigma (St. Louis, MO, USA). All cell culture media and plasticware were procured from Whitehead Scientific (Cape Town, South Africa).

Clinical isolates of CRE, MRSA and VRE were obtained from Lancet Laboratories, Durban, South Africa, with approval BE394/15 from the Biomedical Research Ethical Committee of the University of KwaZulu-Natal. Five reference strains of bacteria, namely *Escherichia coli* ATCC 25922, *Pseudomonas aeruginosa* ATCC 27853, *Enterococcus faecium* ATCC 35667, *Staphylococcus aureus* ATCC 29213 and *Bacillus subtilis* ATCC 6051 were supplied by the American Type Culture Collection (ATCC).

### 4.3. Determination of Minimum Inhibitory Concentrations

MIC values were determined by the broth microdilution method and the significance of the values obtained was interpreted following the guidelines of the Clinical and Laboratory Standards for Antimicrobial Susceptibility Testing. [20]. The effect of human serum on the antimicrobial effect of the molecules tested was determined in a similar way to the MIC method used above in a solution of 50% human serum plus Mueller–Hinton broth.

### 4.4. Cytotoxicity

Cell viability was evaluated using the colorimetric MTT assay [21]. Human hepatocarcinoma (HepG2) cells and human embryonic kidney 293 (HEK-293) cells used in the drug toxicity assays were obtained from Cellonex (Johannesburg, South Africa). Viable HepG2 and HEK293 cells (20,000 cells/well) were seeded into a 96-well microtiter plate (200 µL) and allowed to attach overnight. The cells were exposed to varying concentrations of the peptides for 24 h. After removal of the treatment medium, 20 µL of MTT salt solution (5 mg/mL in 0.1M phosphate buffer saline (PBS)) and 100 µL of complete cell culture medium (CCM) were added to each well. After a 4 h (37 °C) incubation, the MTT salt solution was discarded and 100 μL of dimethyl sulphoxide (DMSO) were added to each well (1 h, 37 °C). The optical density of the solubilized formazan product was measured at 570 nm (reference λ of 690 nm) using a Bio-tek μQuant spectrophotometer (Winooski, VT, USA). The results were reported as percentage of cell viability ($\left(\frac{Absorbance\;of\;treated\;cells}{Absorbance\;of\;control\;cells}\right)\times 100$) versus log concentration of the extract. GraphPad Prism (V5) (La Jolla, CA, USA) was used to obtain the half maximum inhibitory concentration (IC_50_) for each compound.

### 4.5. Time–Kill Curves

Killing curve assays were performed with a starting inoculum of 6 × 10^6^ CFU/mL. Strains were tested against the peptides at concentrations below the MICs. Antimicrobials were added to 5 mL of bacteria in the exponential phase of growth and incubated at 37 °C with shaking. Samples were obtained aseptically at 0, 1, 2, 4, 6 and 24 h, diluted in Ringer 1/4 and plated on TSA for colony counting. The response of microbial strains to a single antimicrobial was determined by lowering logarithms of viable bacteria. In accordance with Lora-Tamayo et al. [22], an antimicrobial was considered active when a reduction of ≥1 log10 relative to the initial inoculum was observed.

### 4.6. Effect on Bacterial Growth Curves

The study of the growth dynamic of the *S. aureus, E. coli* and *P.aeruginosa* strains against CAMPDs were assayed with a starting inoculum of 10^6^–10^8^ CFU/mL in a 10 mL specific falcon tube. The experiment was performed by a real-time reverse-spin bioreactor RTS-1 (Biosan SIA, Riga, Latvia) that applies non-invasive, mechanically driven rotation; thus, the cell suspension is mixed efficiently, mixing and oxygening the culture at 37 °C. Cell growth kinetics were recorded non-invasively in real time from data obtained from a near-infrared optical system, every 15 min.

## Figures and Tables

**Table 1:** SPCDs and their charges.

	Peptide Dendrimer Sequence	Short Name	Residues (Basic/Hydrophobic)	Positive Charges
1	(KLK)_8_(***K***KLK)_4_(***K***KLK)_2_***K***LLKLL-NH_2_	G3KLK-L_2_KL_2_	54 (29/25)	37
2	(KLK)_4_(***K***KLK)_2_***K***LLKLL-NH_2_	G2KLK-L_2_KL_2_	26 (13/13)	17
3	(Ac-KLK)_4_(***K***KLK)_2_***K***LLKLL-NH_2_ ^a^	Ac-G2KLK-L_2_KL_2_	30 ^a^ (13/17)	13
4	(Hx-KLK)_4_(***K***KLK)_2_***K***LLKLL-NH_2_	Hx-G2KLK-L_2_KL_2_	30 ^a^ (13/17)	13
5	(Dd-KLK)_4_(***K***KLK)_2_***K***LLKLL-NH_2_	Dd-G2KLK-L_2_KL_2_	30 ^a^ (13/17)	13
6	(RLR)_4_(***K***RLR)_2_***K***LLRLL-NH_2_	G2RLR-L_2_RL_2_	26 (13/13)	17
7	(OLO)_4_(***K***OLO)_2_***K***LLOLL-NH_2_	G2OLO-L_2_OL_2_	26 (13/13)	17
8	(KLK)_2_***K***LLKLL-NH_2_	G1KLK-L_2_KL_2_	12 (5/7)	7
9	(RLR)_2_***K***LLRLL-NH_2_	G1RLR-L_2_RL_2_	12 (5/7)	7
10	(OLO)_2_***K***LLOLL-NH_2_	G1OLO-L_2_OL_2_	12 (5/7)	7

In blue are the basic residues, positively charged at physiological pH. ^a^ The acyl moieties have been considered as hydrophobic residues.

**Table 2:** MIC and MBC (µg/mL) values of the SCPDs tested on susceptible bacteria.

Amtimicrobial Agents	Gram-Positive									Gram-Negative					
	*S. aureus* ATCC 29213			*B. subtilis* ATCC 6051			*E. faecium* ATCC 35667			*E. coli* ATCC 25822			*P. aeruginosa* ATCC 27853		
	MIC	50% HS *	MBC	MIC	50% HS *	MBC	MIC	50% HS *	MBC	MIC	50% HS *	MBC	MIC	50% HS	MBC
G3KLK-L_2_KL_2_	16	16	16	4–8	8	8	64	64	64	32	32	32	16	16	16
G2KLK-L_2_KL_2_	32	32	32	8	8	8	32	64	32	16	32	32	8–16	16	16
Ac-G2KLK-L_2_KL_2_	64	64	128	8–16	16	16	64	128	128	8–16	16	16	8–16	16	16
Hx-G2KLK-L_2_KL_2_	128	256	128	64	64	128	32	32	64	64	64	64	64	64	64
Dd-G2KLK-L_2_KL_2_	>128	ND	ND	>128	ND	ND	>128	ND	ND	>128	ND	ND	>128	ND	ND
G2RLR-L_2_RL_2_	32	32	32	16	32	16	16	32	16	64	64	64	64	64	64
G2OLO-L_2_OL_2_	32	32	32	32	32	32	8	16	8	32	32	32	32	32	32
G1KLK-L_2_KL_2_	128	>128	128	64	128	64	>128	ND	ND	32	32	32	32	32	32
G1RLR-L_2_RL_2_	16	32	16	32	32	32	4	8	4	8	16	8	16	32	16
G1OLO-L_2_OL_2_	64	64	64	32	32	32	16	16	16	8	8	8	16	16	16
Meropenem	0.25	ND	ND	0.062	ND	ND	<0.25	ND	ND	0.125	ND	ND	1	ND	ND

* MIC values in the presence of 50% human serum.

**Table 3:** MIC (µg/mL) values of the SCPDs tested on MRSA vancomycin susceptible isolates.

Isolates	G3KLK -L_2_KL_2_	G2KLK -L_2_KL_2_	Ac-G2KLK -L_2_KL_2_	Hx-G2KLK -L_2_KL_2_	G2RLR-L_2_RL_2_	G2OLO -L_2_OL_2_	G1RLR-L_2_RL_2_	G1OLO-L_2_OL_2_	Van	Amp
B11970	32	64	128	64	32	64	16	64	1	>512
P10781	16	64	128	64	64	64	32	64	1	>512
P10747	16	64	128	64	64	64	32	64	1	>512
S37938	32	64	128	64	32	64	32	64	1	>512
S18155	32	64	128	64	64	64	16	64	1	>512
B13178	32	64	128	64	32	64	16	64	1	>512
440260	32	64	128	64	32	64	16	64	1	>512
S18970	32	64	128	64	32	64	16	64	1	>512
P11520	16	64	128	64	32	64	16	64	1	512
T5683	32	64	128	64	64	64	16	64	1	>512

**Table 4:** MIC (µg/mL) values of the SCPDs tested on VRE, *E. faecium* isolates.

Isolates	G3KLK -L_2_KL_2_	G2KLK -L_2_KL_2_	Ac-G2KLK -L_2_KL_2_	Hx-G2KLK -L_2_KL_2_	G2RLR -L_2_RL_2_	G2OLO -L_2_OL_2_	G1RLR-L_2_RL_2_	G1OLO-L_2_OL_2_	Van
951245262	16	64	64	128	16	32	64	16	>128
951234856	32	32	64	64	32	64	32	16	>128
951208931	16	32	128	64	16	32	32	32	>128
938636470	32	32	64	128	16	64	64	16	>128
938666613	16	32	128	64	16	32	32	32	>128
938600912	16	32	64	64	16	32	32	16	>128
938072607	32	32	128	64	32	64	32	32	>128
944414000	32	32	128	64	16	64	32	32	>128
945530665	32	32	128	64	16	32	32	32	>128
U43821	32	32	128	64	16	32	32	32	>128

**Table 5:** MIC (µg/mL) values of the SCPDs tested on CRE, *E. coli* isolates.

Isolates	G3KLK -L_2_KL_2_	G2KLK -L_2_KL_2_	Ac-G2KLK -L_2_KL_2_	Hx-G2KLK -L_2_KL_2_	G2RLR-L_2_RL_2_	G2OLO -L_2_OL_2_	G1RLR-L_2_RL_2_	G1OLO-L_2_OL_2_	Merop
VIM-1 BM-12	>256	64	64	128	64	64	8	8	>32
NDM4-FEK	256	32	64	64	32	32	8	8	>32
KPC L2l	32	32	64	64	64	32	8	8	16
OXA-48 501	16	32	32	128	64	16	16	16	2
IMP JAP	64	32	64	>256	64	8	8	8	8

## Data Availability

Not applicable.

## Supplementary Materials

The following are available online at https://www.mdpi.com/article/10.3390/antibiotics10060695/s1. **Figure S1**. 24 h Time-kill curves against *E. coli* for G2RLR-L_2_RL_2_, G2OLO-L_2_OL_2_, G1KLK-L_2_KL_2_, and G1OLO-L_2_OL_2_. **Figure S2**. HPLC chromatogras and Mass Spectra of all compounds synthesized.
