# Supplementary material for: Super-Cationic Peptide Dendrimers—Synthesis and Evaluation as Antimicrobial Agents
Source: Antibiotics (Basel). 2021 Jun 10;10(6):695. doi: 10.3390/antibiotics10060695 (PMC8228121; doi:10.3390/antibiotics10060695)
Supplement: Supplementary file 1 [file antibiotics-10-00695-s001.zip › antibiotics-1245431-supplementary.pdf]

Super-Cationic Peptide Dendrimers. Synthesis and Evaluation as Antimicrobial Agents.

Estelle J. Ramchuran[1,2,$], Isabel Pérez-Guillén [3,$], Linda A. Bester [1], René Khan [4], Fernando Albericio [2,5,6*], Miguel Viñas [3*], and Beatriz G. de La Torre [7*]

[1] Biomedical Resource Unit, School of Laboratory Medicine and Medical Sciences, College of Health Sciences, University of KwaZulu-Natal, Durban 4000, South Africa
[2] Peptide Sciences Lab oratory, School of Chemistry and Physics, University of KwaZulu-Natal, University Road, Westville, Durban 4001, South Africa
[3] Lab. Molecular Microbiology & Antimicrobials. Department of Pathology and Experimental Therapeutics, Medical School-IDIBELL, University of Barcelona, Hospitalet, Barcelona 08907, Spain
[4] Discipline of Medical Biochemistry, School of Laboratory Medicine and Medical Science, University of KwaZulu-Natal, Durban, South Africa.
[5] Institute for Advanced Chemistry of Catalonia (IQAC-CSIC), 08034-Barcelona, Spain
[6] CIBER-BBN, Networking Centre on Bioengineering, Biomaterials and Nanomedicine, and Department of Organic Chemistry, University of Barcelona, 08028-Barcelona, Spain
[7] KRISP, College of Health Sciences, University of KwaZulu-Natal, Westville, Durban 4001, South Africa
$ These two authors contribute equally to the paper.
* Correspondence: garciadelatorreb@ukzn.ac.za, mvinyas@ub.edu, albericio@ukzn.ac.za

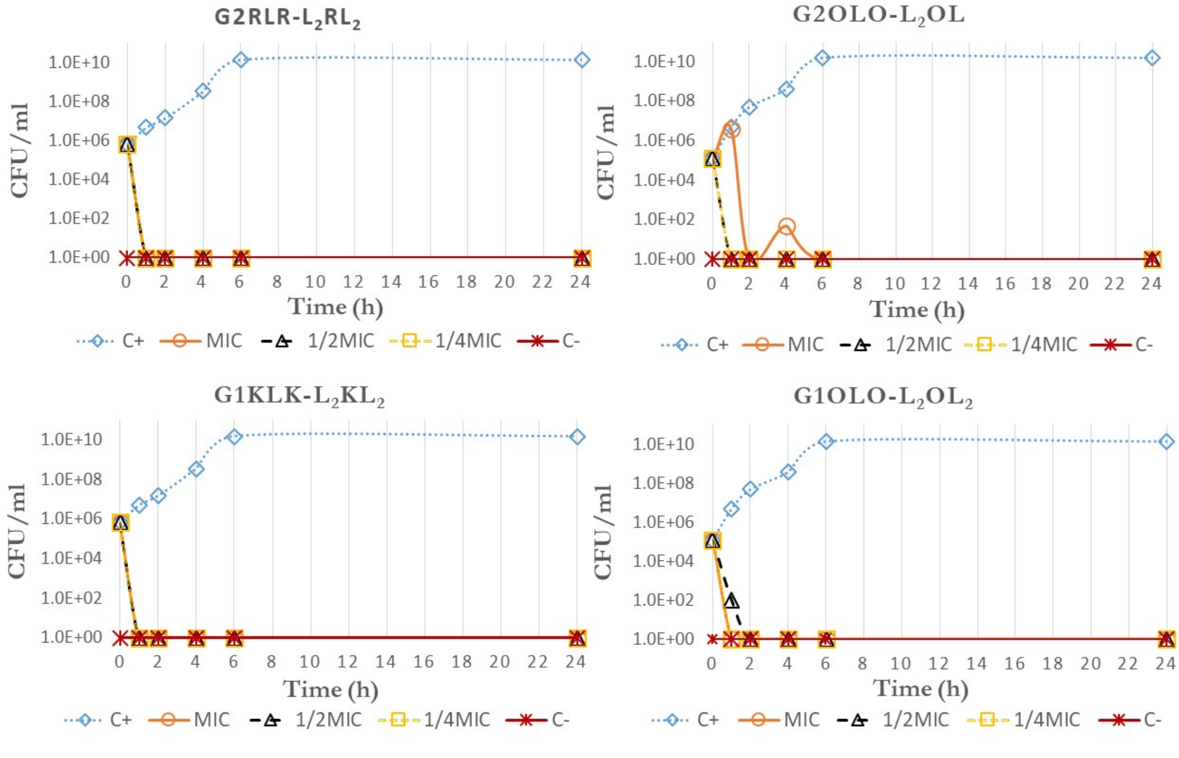

Figure S1. 24 h Time-kill curves against *E. coli* for G2RLR-L_2RL_2, G2OLO-L_2OL_2, G1KLK-L_2KL_2, and G1OLO-L_2OL_2

Analytical LC-MS was performed in aThermo Scientific™ UltiMate™ 3000 Standard Binary System, ISQ™ EC Single Quadrupole. Buffer A: 0.1% formic acid in H_2O; buffer B: 0.1% formic acid in CH_3CN; Flow: 1.0mL/min., UV detection=220 nm; Mass were registered in positive mode; Column: Phenomenex Luna C18 3.6 µm, 4.6 × 150 mm column, 30 °C. The method is specified for each chromatogram.

G3KLK-L_2KL_2: MW=6668.21.

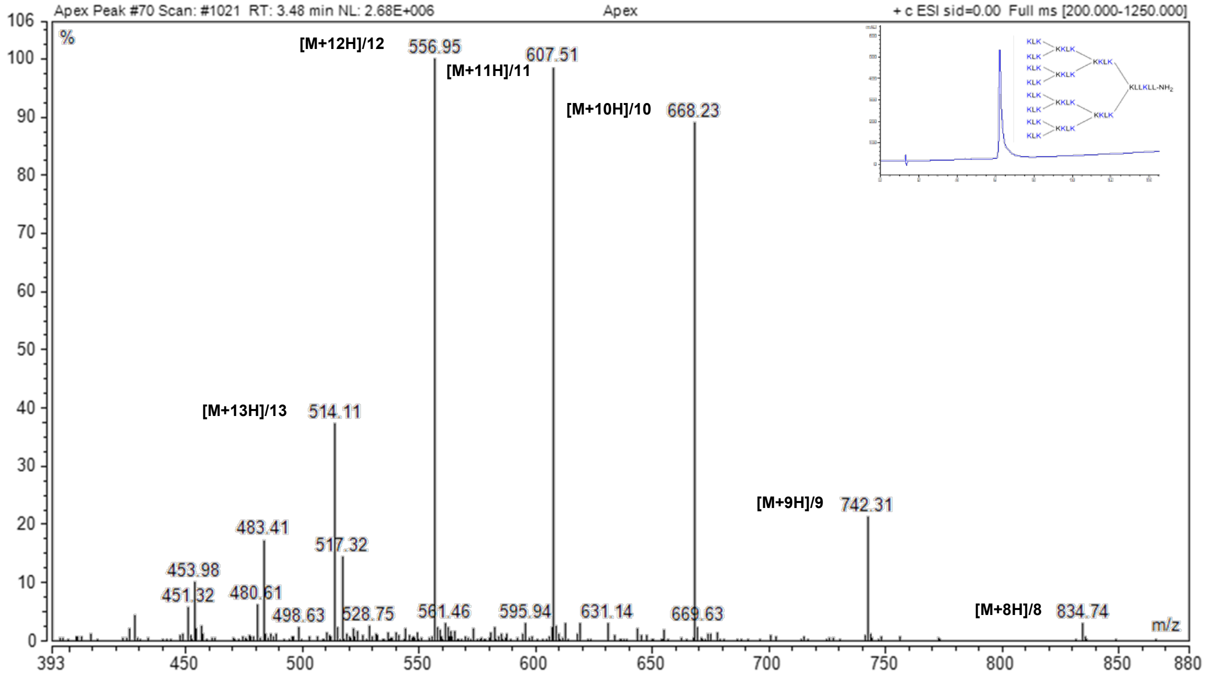

Method: 20% to 40% of B into A in 15 min.

G2KLK-L$_2$KL$_2$: MW=3199.43

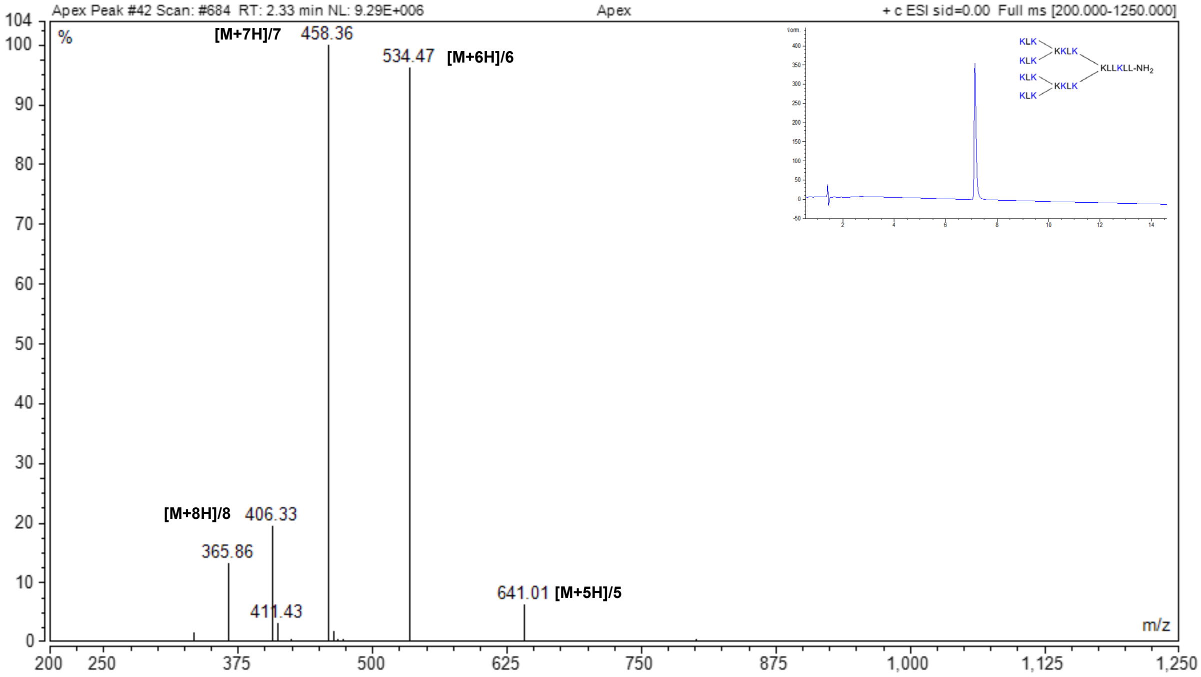

Method: 10% to 60% of B into A in 15 min.

Ac-G2KLK-L$_2$KL$_2$: MW=3367.58

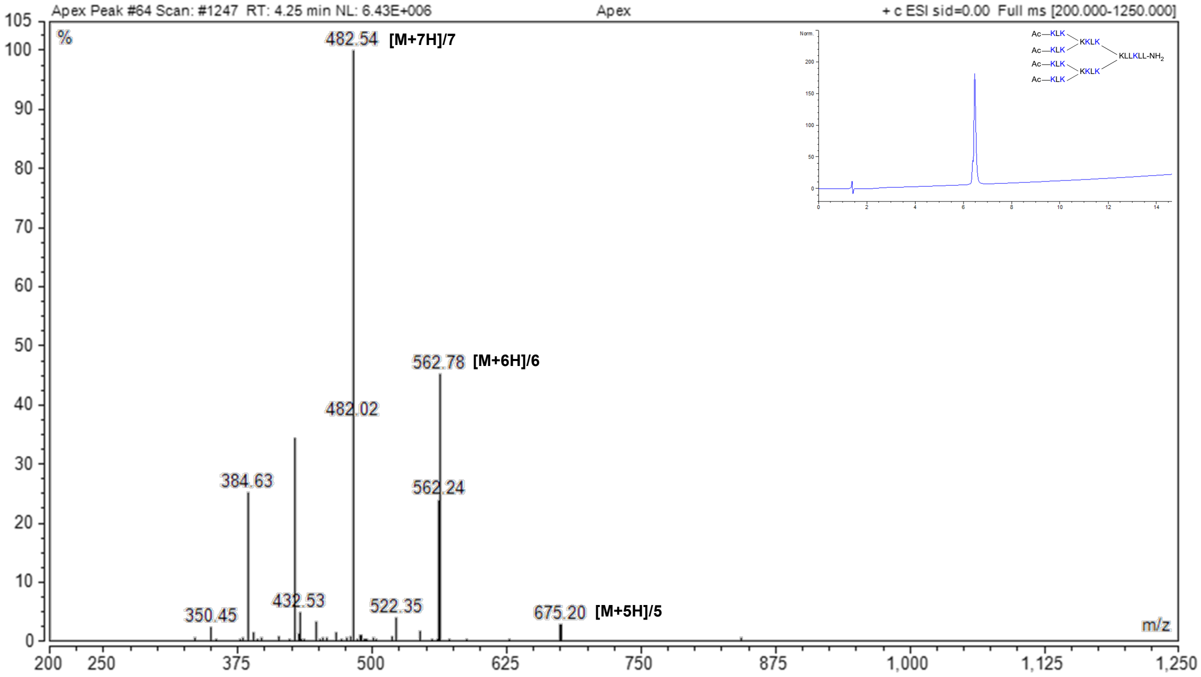

Method: 20% to 60% of B into A in 15 min.

Hx-G2KLK-L$_2$KL$_2$: MW=3592.68

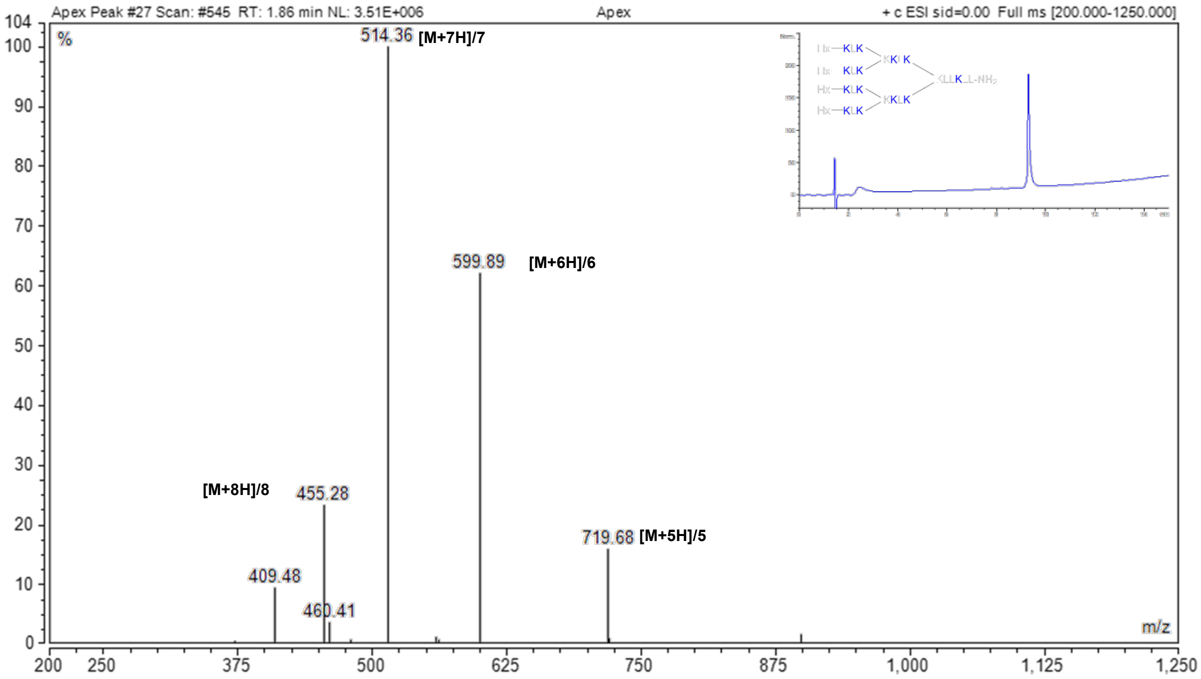

Method: 10% to 70% of B into A in 15 min.

Dd-G2KLK-L$_2$KL$_2$: MW=3928.66

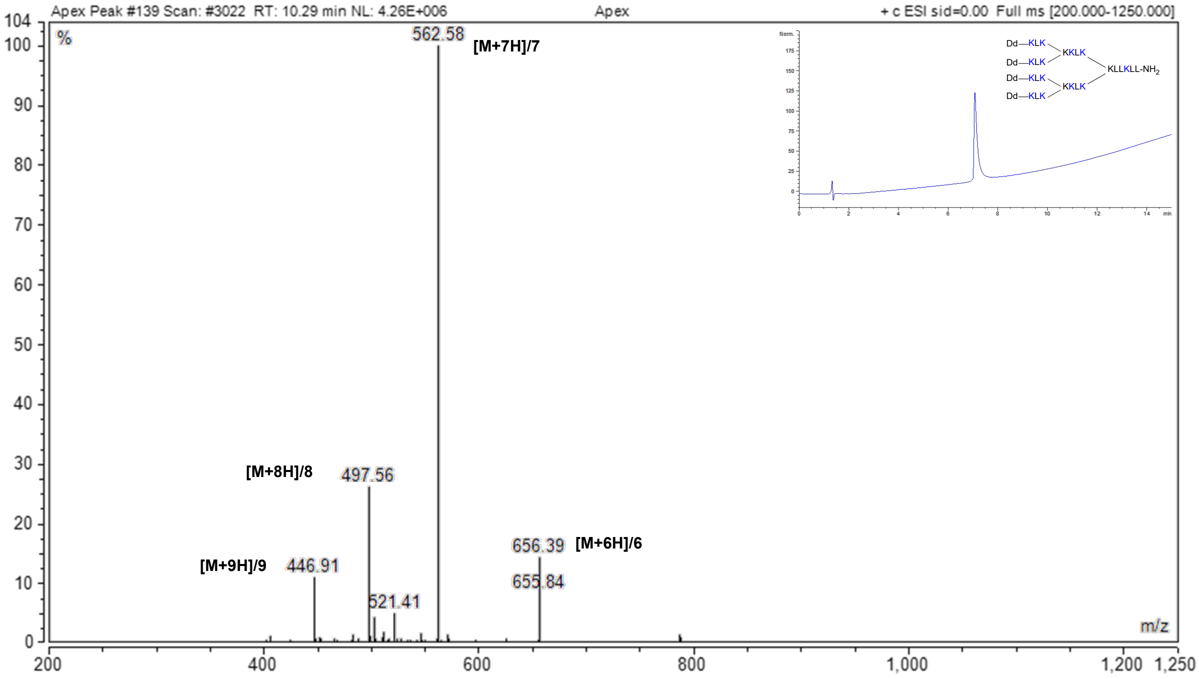

Method: 10% to 70% of B into A in 15 min.

G2RLR-L$_2$RL$_2$: MW=3562.61

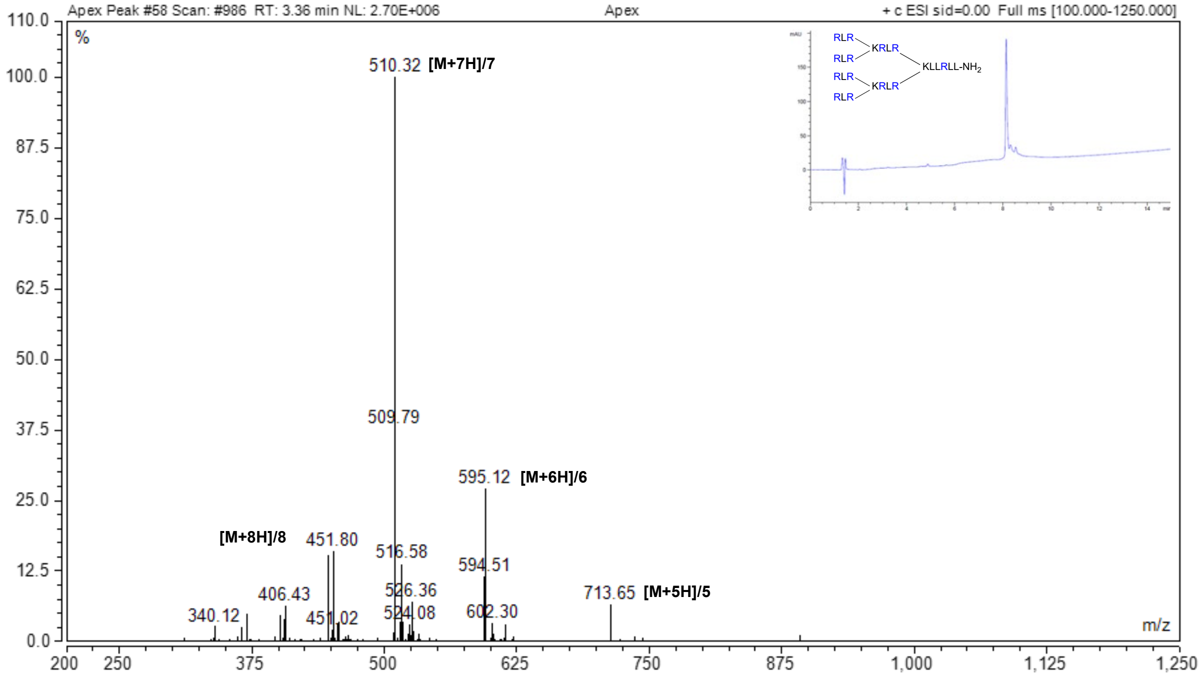

Method: 10% to 60% of B into A in 15 min

G2OLO-L$_2$KL$_2$: MW=3017.08

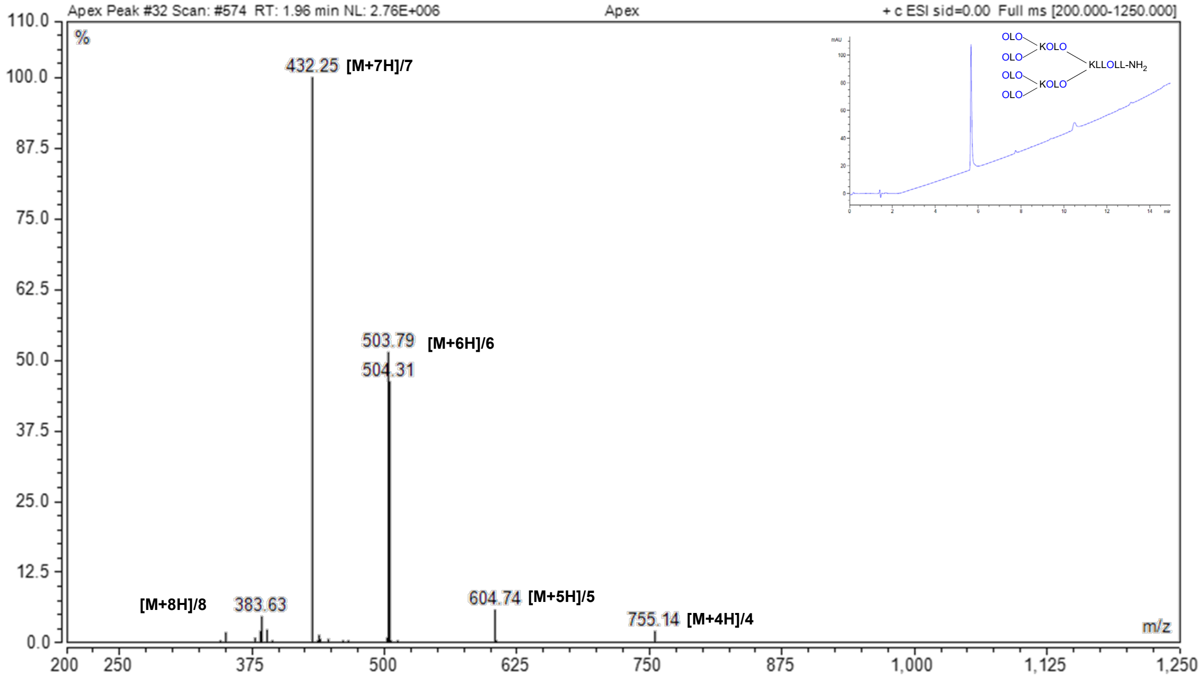

Method: 10% to 70% of B into A in 15 min

G1KLK-L$_2$KL$_2$: MW=1465.04

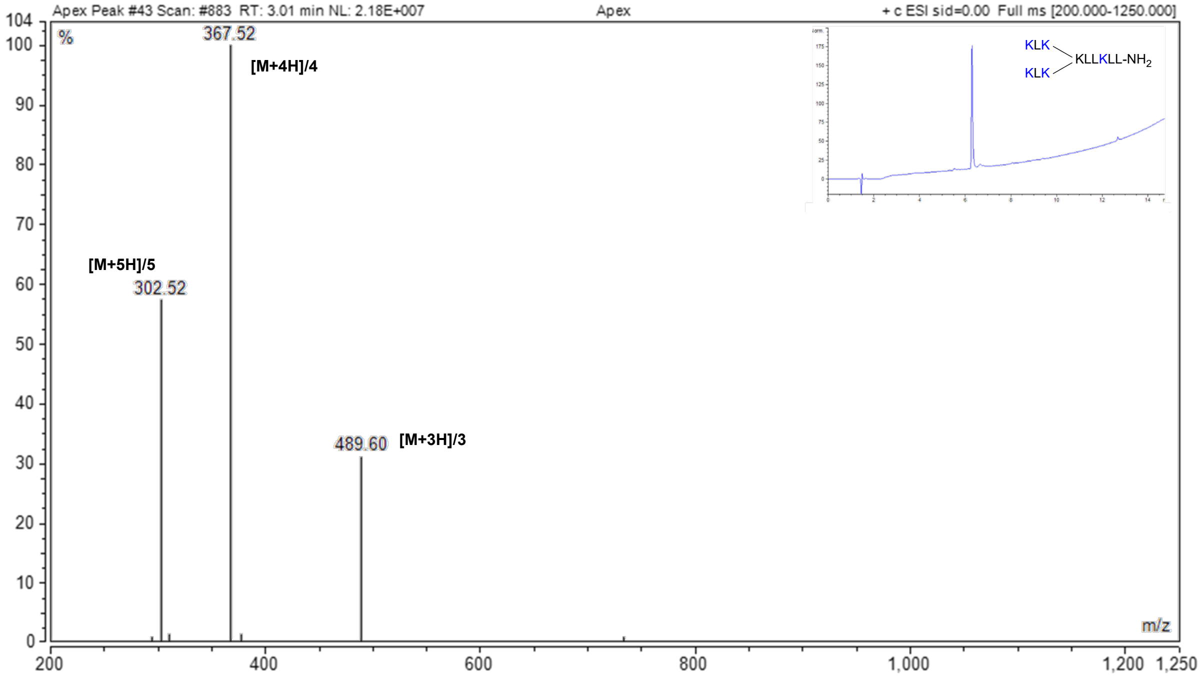

Method: 5% to 95% of B into A in 15 min.

G1RLR-L$_2$KL$_2$: MW=1604.10

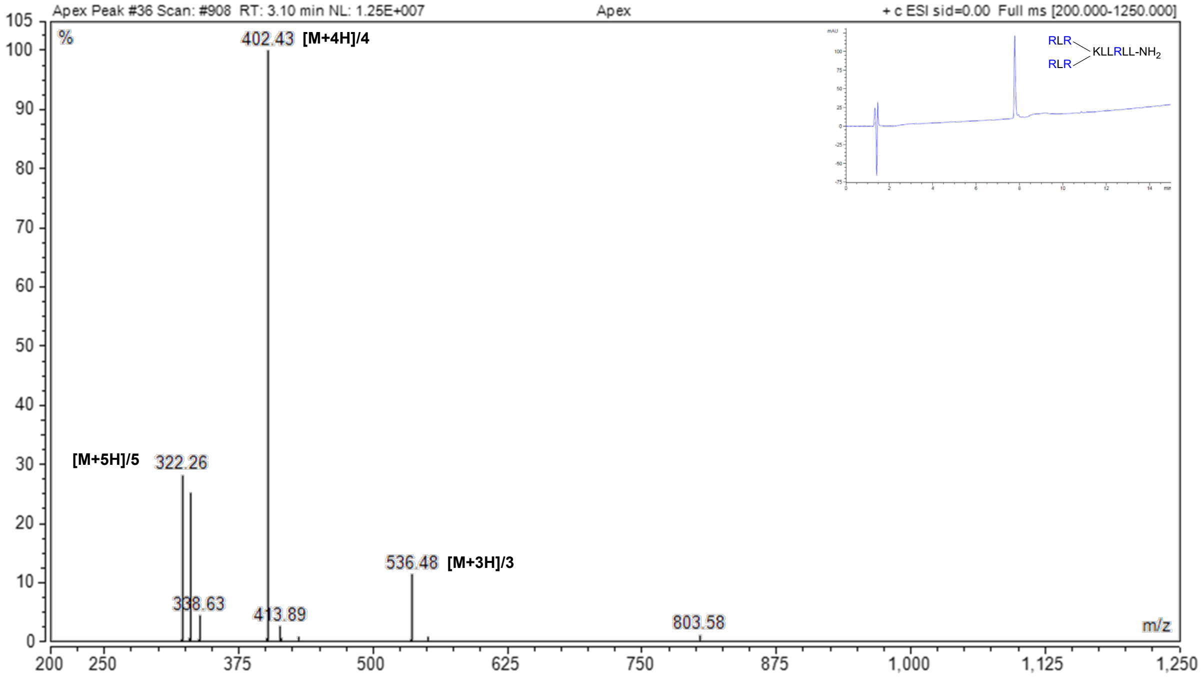

Method: 10% to 60% of B into A in 15 min.

G1OLO-L_2KL_2: MW=1394.02

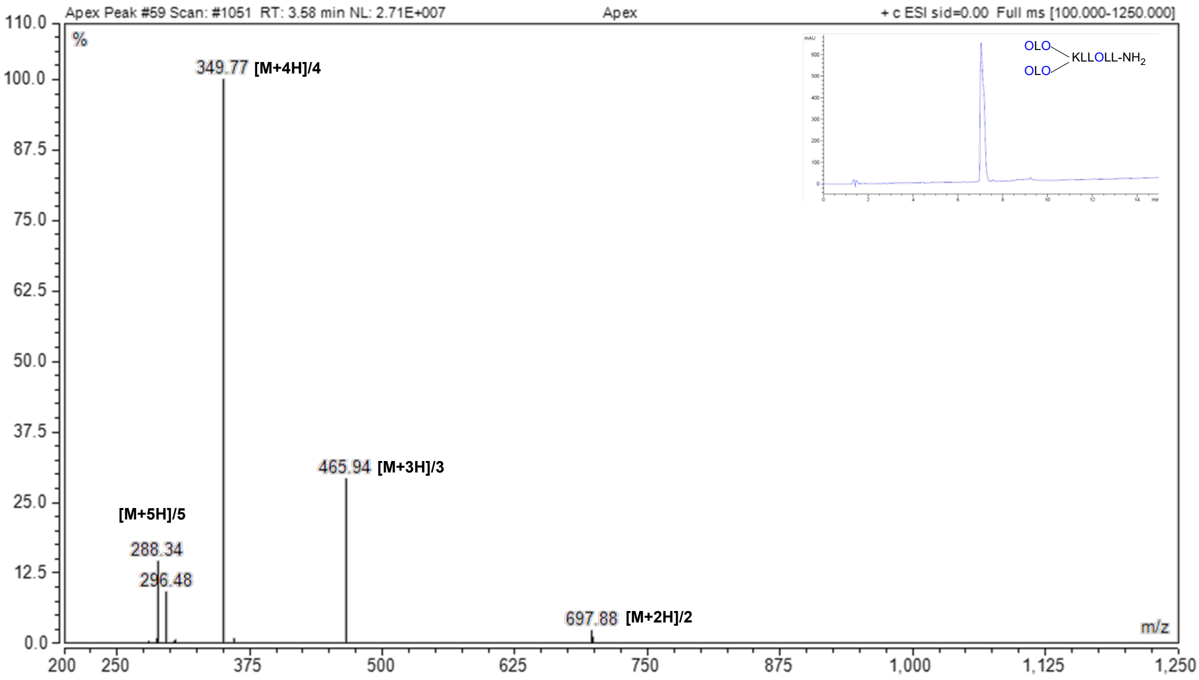

Method: 10% to 60% of B into A in 15 min.
